# Influence of Public Sports Services on Residents’ Mental Health at Communities Level: New Insights from China

**DOI:** 10.3390/ijerph20021143

**Published:** 2023-01-09

**Authors:** Liu Lu, Wei Wei

**Affiliations:** 1 College of Physical Education, Chengdu Sport University, Chengdu 610041, China; 2 School of Physical Education and Sports Science, South China Normal University, Guangzhou 510630, China

**Keywords:** public sports services, mental health, two-factor model, multi-level regression model

## Abstract

It is generally believed that sports play an important role in healing and boosting mental health. The provision of public sports services is important for enhancing residents’ physical fitness and mental health, and for promoting their satisfaction with government public services. To build and strengthen a high-quality sports service-oriented society, it is important to explore whether community public sports services influence residents’ mental health. To explore this phenomenon, the study gathered data from China and employed multi-level regression models to meet the study objective. The results show that the residents’ age difference is 0.03, and the average daily exercise time is 0.02, which is significantly correlated with residents’ mental health. The results show that the lower the availability and greening of sports facilities, and the fewer rest facilities there are, the higher the mental distress of residents may be. Conversely, the improvement of the greening and availability of sports facilities can facilitate the promotion of residents’ mental health levels. Moreover, it was found that the mental health of residents is mainly and positively affected by the cleanliness of sports facilities. The street environment affects mental health and is attributed to the damage to sports facilities. Neighborhood communication also improves residents’ mental health, and trust between neighbors has the greatest impact on reducing mental distress. Finally, the study proposes that the government should propose strategies to optimize the provision of community public sports services in the study area to boost both social and mental health benefits.

## 1. Introduction

The domestic residential community refers to the community in which people live in a certain area in a city and is the most basic social unit in which people live. It has two basic conditions. One is regional; this mainly includes residents, shops, and schools. The second is the common needs of life, and cultural identity [1]. Public sports services refer to the provision of related sports products and services for the public [2]. Public sports services exist to promote and develop sports services within the service-oriented government functions, to meet the needs of community residents for various public sports activities. It guarantees sports rights for residents [3]. Community sports services are public sports services that specifically refer to the sports needs of social residents and are provided by various organizations and individuals funded by the government. It is a public service rather than a profit-making service [4]. It is characterized by mutual assistance and welfare. The target is to improve the health of community residents. It is a sports service activity which is carried out based on theoretical sports knowledge [5].

In the prevailing literature, there have been many studies exploring the impact of the provision of community public sports services on residents’ mental health. For example, Chen (2020) examined the impact of community sports provision on social inclusion and public health using micro data from household surveys in the rural areas of eight Chinese provinces. The results found that community sports provision partially and effectively promotes health in rural areas, and reduces the risk of disease [6]. Kim (2020) used structural equation model analysis to show that older adults who were aware of social cohesion were likely to participate in light, moderate, and vigorous leisure-time physical activity, which supported mental health [7]. Dixon (2019) also analyzed the impact of football on mental health resilience in adult men [8]. However, there are many other studies on public sports services. Most of them only brought to light issues such as the status quo of the provision of public sports services. Further and specific research on the masses, such as the research on residents’ satisfaction with public sports services in urban communities, is rare. The results show that football culture has a practical application value in improving men’s mental health. Many issues have not been given enough attention. Therefore, this study innovatively explores the current situation of community public sports service provision. Moreover, the study also explores whether or not residents are satisfied with public sports services. The study used multi-level regression models and simple mediation models to obtain deeper insights. The influence and correlation of various factors regarding residents’ mental health are also explored.

The remainder of the article is organized as follows. The theoretical basis is briefly elaborated in Section 2. Section 3 and Section 4 describe the research methods and report and discuss the findings. Section 5 concludes the study with policy implications and gives the limitations of the study.

## 2. Theoretical Framework

The study initially discusses the “construction” model. The “construction” model is analyzed from the perspective of environmental cognitive psychology, and the principle that the environment affects mental health [9]. The core is the belief that each individual develops a personal construct as they grow up. Each individual’s long-term understanding and evaluation of people, objects, and events in the surrounding environment form their collection of ideas [10]. Moreover, the mental health of an individual refers to the mature personal structure and clarification of the personal ideological structure through rational thinking, so the individual coexists harmoniously with the surrounding environment [11]. The “construction” model believes that the influence of the surrounding environment on an individual’s mental health is expressed by the individual’s subjective cognition, which is also the basis of mental health research [12].

And the other notion is a two-factor model of mental health. The traditional theoretical model of mental health regards subjective well-being and mental distress as two opposite extremes [13]. The two-factor model of mental health starts from a multi-dimensional perspective and believes that an individual’s mental health is judged from both the positive state and the negative state of mental health [14]. The two-factor model believes that mentally healthy individuals not only need to rid themselves of their mentally unhealthy state but also need to obtain a positive mentally healthy state. The two states of an unhealthy mental state and a positive mental health state are independent and unified. Based on this notion, subjective well-being and mental distress symptoms are taken as two classification criteria [15], and the population is divided into four categories including (1) complete mental health, (2) partial mental health, (3) partial mental distress and (4) total mental distress.

People who are completely mentally healthy can express their emotions effectively and have good social adaptation functions. Although some mentally healthy people do not have the mental pain required for a psychiatric diagnosis, they also lack the positive energy required for mental health, so they may have mental problems in the future [16]. Some people with mental distress will have some symptoms of mental illness, but they can improve their state through their positive energy. People with complete mental distress have high levels of mental distress and low subjective well-being [17]. The two-factor model of mental health reveals that in the development of mental health, it is necessary to treat people with mental problems in time to improve their healthy mental state and prevent the occurrence of adverse mental problems [18]. The model establishes a bi-level understanding of residents’ mental health. From the perspective of the impact of the provision of community public sports services on residents’ mental health, the provision of high-quality community public sports services can increase happiness, enhance residents’ positive emotions, and reduce the probability of residents suffering from mental distress. 

Therefore, research on the impact of community public sports service provision on mental health must be divided into positive and negative levels to quantify mental health outcomes [19].

## 3. Methodology

### 3.1. Data Sources

To meet the study objectives, data were collected from Guangzhou city of Guangdong province of China. A total of 180 questionnaires were distributed in five communities of Guangzhou, and 168 questionnaires were received. The study was conducted by random sampling from June to October 2021. The questionnaire was issued both online and offline. The questionnaire is distributed offline within the five communities, and it was issued online through the questionnaire website (https://www.wenjuan.com/ (accessed on 13 October 2022)). At the same time, to preserve the real intention of the respondents, the study was performed anonymously. After removing invalid questionnaires with missing data, 159 valid questionnaires were retained for empirical analysis. Among 159 valid questionnaires, 66 were females and 93 were male respondents. The effective utilization rate of the questionnaire was 88.33%. In addition, 35 residents were re-investigated to test the reliability of this questionnaire. The questionnaire was measured on a Likert scale, and the data of this questionnaire were analyzed using SPSS software (manufacturer, city, country). The P-value of the retest reliability coefficient obtained was less than 0.01, which shows that the questionnaire met the reliability requirements.

Furthermore, the coefficient of internal consistency is one of the main indicators of questionnaire reliability of the test scale, and there are relatively unified opinions on its classification criteria. According to the relevant theory of statistics, the reliability coefficient of the scale or test is above 0.90, indicating that the scale is very reliable. By testing the reliability coefficients of each dimension and the whole scale of the community public sports service quality evaluation scale, it was found that the coefficient of internal consistency of the five dimensions is above 0.7, and the consistency coefficient of the whole scale is above 0.9, illustrating that the scale is effective and reliable, and can be used as a research tool. The exploratory factor analysis is used to test the structural validity of the scale for evaluating the quality of community public sports services. The approximate Chi-square of the scale is 298.699, which reaches a significant level, indicating that the scale had good structural validity.

### 3.2. Empirical Estimation

The study initially employs the multi-level regression model. The model typically deals with repeated measurements of the same individual. There is a correlation between the data measured repeatedly by the same individual, so ordinary linear regression is no longer applicable [20]. The mental health of residents will be affected by multiple levels of personal attributes and external factors. The traditional single-level model research method only focuses on the level changes of residents’ attributes or external factors, ignoring the differences between residents or external factors. The multifactorial influencing mechanisms of health status obtained are often not convincing [21]. 

The effectiveness of community public sports services is affected by multiple factors. Therefore, when evaluating the quality of community public sports services, the characteristics, content, and the ways these services are realized need to be fully considered. The principle of comprehensiveness is to be followed to understand the evaluation of public sports service quality as a whole, and to take into account all of the important factors on public sports service quality. The index system can fully reflect the real situation of public sports service quality in communities. In addition, during the construction of the public sports service quality evaluation system, ensuring the integrity and accuracy of the indicators but also the operability of indicators was taken into consideration.

The multi-level model can separate the influence of different characteristic variables, so it can test the influence of each level and the contribution of each level to explain the difference of the dependent variable [22]. The model is as shown below:

(1) $$Y_{ij}=\alpha +\beta W_{j}+\gamma X_{ij}+\mu_{j}+\varepsilon_{ij}$$

In Equation (1), *Y_ij_* means that *i* = 1, 2, …, *n* exists at the first level. These *n* individuals belong to *j* = 1, 2, …, *n* community public sports facilities supply on the second floor. *γ* represents the coefficient at the individual level. *α* and *β*, respectively, represent the coefficients of the supply level of community public sports facilities and are fixed effects [23]. *X* is the independent variable at the individual level (such as the residents’ gender, age, education level, and other individual attributes). *W* is the independent variable of the supply level of community public sports facilities. *ε* is a random effect at the individual level. *μ* is the random effect of the supply level of community public sports facilities [24]. Equation (1) is:

(2) $$E_{\left(ij\right)}=0,{Var}_{\left(\varepsilon_{ij}\right)}=\sigma^{2}$$

(3) $$E_{(\mu_{j)}}=0,{Var}_{\left(\mu_{j}\right)}=\tau^{2}$$

In Equations (2) and (3), ${Var}_{\left(\varepsilon_{ij}\right)}$ = *σ*^2^ represents the variance of the individual level, and ${Var}_{\left(\mu_{j}\right)}$ = *τ*^2^ represents the variance of the level of provision of community public sports facilities [25]. The influence of different levels of variables on the mental health of residents is obtained by comparing the model with only control variables in the multi-level linear regression model and the model with the supply of community public sports facilities [26]. The multi-level regression model steps are as follows, (1) Construct model I, containing only control variables, examine the control variables and judge the applicability of the multi-layered model. (2) Construct a model II that includes the supply of community public sports facilities, and explore its total impact on residents’ mental health. (3) Construct a model III that includes the provision of community public sports facilities, and explore the total impact of its independent variables on residents’ mental health. (4) Identify the impact of the provision of public sports facilities in different communities on mental health in the established model II and (5) analyze the influence degree of the three characteristics on mental health. The above-mentioned three models must be constructed to explore the overall impact of the provision of community public sports facilities on residents’ mental health and the degree of impact of three characteristics on mental health [27]. Model I contains control variables. Model II and model III encompass the provision of community public sports facilities [28].

The study also employed mediation analysis. It is important to observe whether the activity environment around sports facilities plays a mediating role between the provision of community public sports facilities and mental health [29]. The activity environment around sports facilities plays a mediating role between the provision of community public sports facilities and mental health [30]. The internal mechanism of its impact on mental health can be explored further by testing the mediating effect of the activity environment around sports facilities [31]. The mediation model can be expressed as:

(4) $$Y=\alpha_{0}+\alpha_{1}X+\varepsilon$$

(5) $$M=\beta_{0}+\beta_{1}X+\mu$$

(6) $$Y=\gamma_{0}+\gamma_{1}X+\gamma_{2}M+\vartheta$$

In Equations (4) and (6), *X* represents the independent variable, *Y* represents the dependent variable, and *M* represents the mediator variable. *ε*, *μ*, and *ϑ* represent random error terms. When the mediation effect model is used, the first step is to test the regression coefficient *a*_1_ of the dependent variable *X* and the independent variable *Y* [32]. When the regression coefficient cannot be ignored, it is necessary to test the regression coefficient *a*_2_ between the mediating variable *M* and the independent variable *X*. Meanwhile, the regression coefficients *a*_3_ and *a*_4_ of the dependent variable *Y* on the independent variable *X* and the mediator variable *M* are tested. If the regression coefficients *a*_3_ and *a*_4_ are also not negligible and *a*_3_ < *a*_1_, there is a mediation effect [33]. The influence of some variables is not consistent and will vary with the changes in the adjustment variables such as the age, gender, and income of the residents [34,35]. Therefore, a mediation effect model with adjustment variables is used to explore the deep role of mediator variables. The improved mediation effect model is composed of the following three equations [36].

(7) $$Y=\alpha_{0}+\alpha_{1}X+\alpha_{2}U+\alpha_{3}U*X+\alpha_{4}Z+\varepsilon$$

(8) $$M=\beta_{0}+\beta_{1}X+\beta_{2}U+\beta_{3}U*X+\beta_{4}Z+\epsilon$$

(9) $$Y=\delta_{0}+\delta_{1}X+\delta_{2}U+\delta_{3}U*X+\delta_{4}M+\delta_{5}U*M+\delta_{6}Z+\mu$$

In Equations (7) and (9), *X* represents the independent variable, *Y* represents the dependent variable, *M* represents the mediator variable, *U* is the adjustment variable, and *ε*, *μ*, and *ϑ* represent random error terms. In the study of the supply of community public sports facilities on residents’ mental health, the mediating effect caused by the influence of moderator variables can be represented by the combination of parameters (*β*_1_ + *β*_3_*U*)(*δ*_4_ + *δ*_5_*U*). Therefore, in the final empirical test, the focus is on the degree of significance of the variables in Equations (8) and (9), and the important coefficients in these two equations are shown [37].

## 4. Results and Discussion

### 4.1. Respondents’ Summary Characteristics 

**Figure 1:**
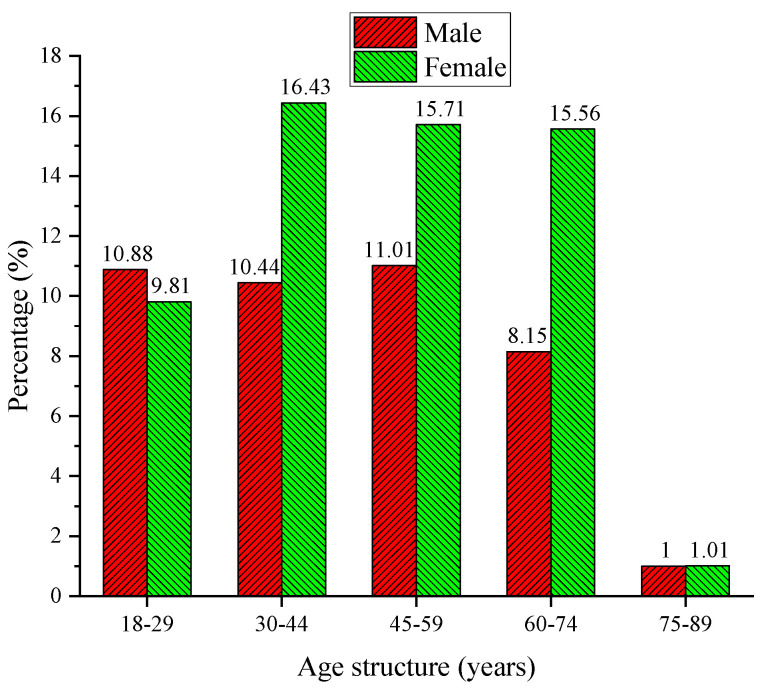
Gender and age composition of residents.

The gender and age composition of residents are shown in Figure 1. As shown in Figure 1, the gender ratios of the surveyed residents are 41.48% and 58.52%, respectively, and the gender ratio is in line with the overall gender ratio of the city. Age distribution of the residents surveyed indicates that the largest proportions are those aged 30–44 and 45–59, which are 26.87% and 26.72%, respectively. In addition, 60–74 year-olds and 18–29 year-olds account for 23.71% and 20.69%. The results imply that the distribution of the number of people in different age groups is different, and there is a big difference. It can be seen that the number of males between the ages of 45 and 59 is the largest, and the number of females between the ages of 30 and 44 is the largest.

**Figure 2:**
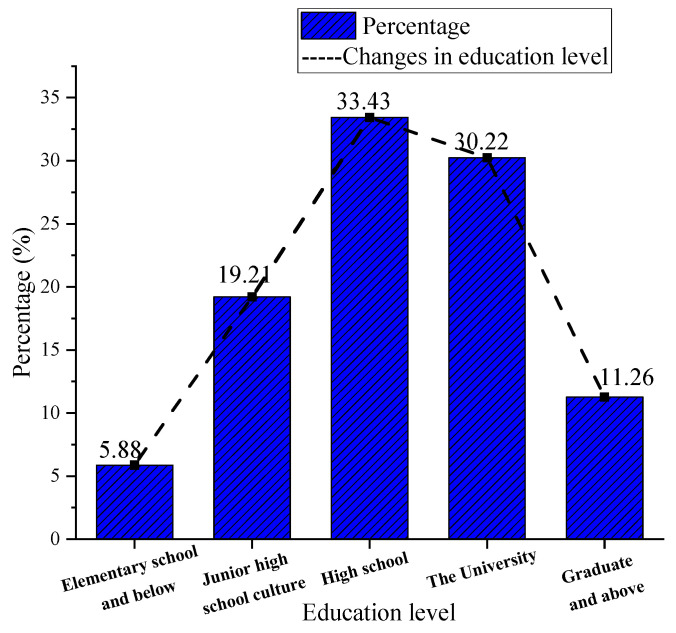
Distribution of the educational level of the surveyed residents.

The study also revealed the educational level of the surveyed residents. As shown in Figure 2, the highest proportion of people with a high-school education is 33.43%. Those with a university degree account for 30.22%. Changes in educational attainment conform to a normal distribution curve, proving the reliability of the data. It can be seen that having a degree is very important for one’s career development. Through the training of professional background knowledge in various universities, individuals can master the skills needed for career development. The topic of this study is the impact of public sports services on the mental health of community residents. Through studying the educational level of the respondents, the factors affecting the mental health of community residents can be further analyzed and explained.

**Figure 3:**
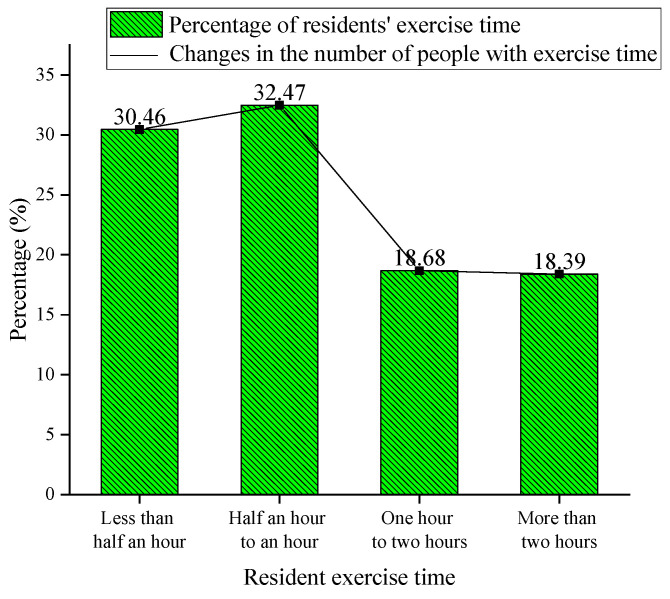
Distribution of the number of people surveyed for exercise time.

The analysis of the average daily exercise time of the surveyed residents is shown in Figure 3. According to this figure, it is shown that the percentage of people who exercised for 30–60 min is the highest, at 32.47%. Those who exercised for less than half an hour accounts for 30.46%. Those who exercised for 1–2 h and more than two hours account for 18.68% and 18.39%, respectively. Therefore, most people go out to exercise for some time every day. It can be seen that the number of people who exercise for half an hour is the largest, followed by those who exercise for less than half an hour and the number of people who exercise for more than two hours is the lowest.

### 4.2. Current Status of Public Services in the Study Area

The study also provided the current situation of the provision of community public sports facilities in the study area as shown in Table 1. Table 1 indicates that different communities also have differences in the configuration of public sports facilities. For example, some communities do not have indoor gyms or table tennis courts. Some community public sports facilities cannot be maintained and updated, and there is an aging situation. This situation is mainly related to the perfection of the management system of each community’s public sports facilities.

The current provision situation of community public sports activities is shown in Table 2. According to the survey and Table 2, community public sports activities are mainly carried out in parks and public sports facilities in the community. The survey data shows that most community activities are small activities. Most of the small and medium-sized events with 50 to 150 people are held every year, such as square dance competitions, followed by medium and long-distance running.

### 4.3. Mental Health Characteristics

**Figure 4:**
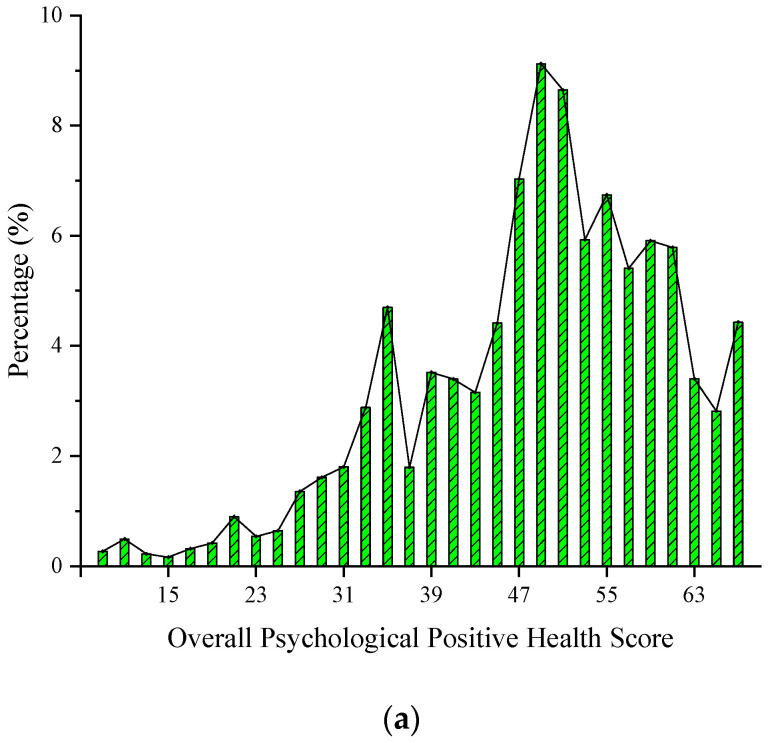

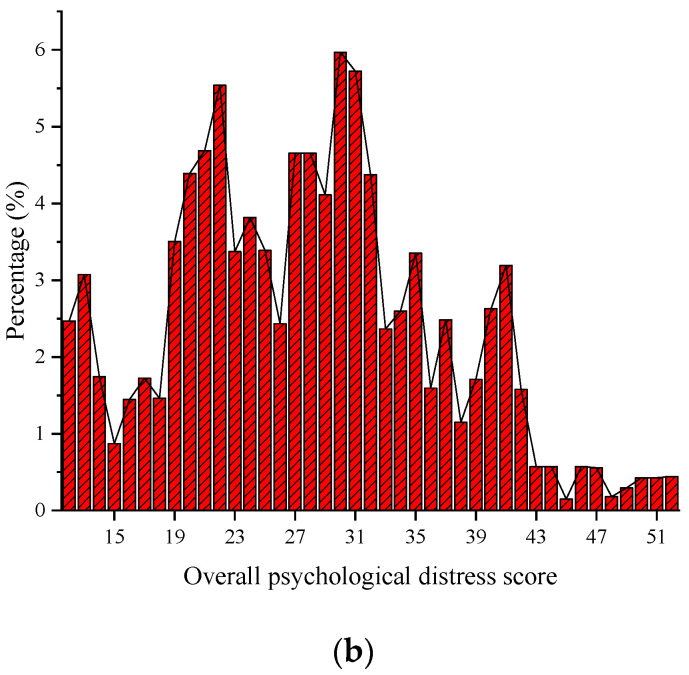
Histogram of positive mental health and mental distress scores of surveyed residents. (**a**) Distribution map of overall mentally positive health scores. (**b**) Distribution map of overall mental distress scores.

According to previous studies, the high and low critical values of mental distress scores are 22 and 10 points, respectively. When the score is between 10 and 15, it is classified as a low-risk group of mental distress. Those scores in the 16–21 range are considered to be at moderate risk. A score of 22–32 is a high-risk group. Scores above 33 are very high-risk groups. Positive psychology scores are the same as above. According to the descriptive statistics of the survey data, the mental distress and positive mental health scores of the residents of the five surveyed communities are shown in Figure 4. Figure 4a implies that the overall level of positive mental health of the surveyed residents is good. From Figure 4b and the high and low critical values of mental distress scores, 74.9% of the risk groups are higher than the critical value of the high score, of which 26.87% are extremely high, indicating that a considerable number of residents are still at a high risk of suffering from mental distress-related diseases. From the perspective of the overall image change trend, both of them are in the normal distribution. It can be seen that the overall mental health level of community residents is between 47 and 55 points, while the overall mental stress score of residents is between 19 and 31.

### 4.4. Differences in the Mental Health Status Based on Resident Characteristics

The grouping variables are the gender, age, educational level, and average daily exercise time of the surveyed residents. The difference in its variable scores was tested to obtain the situation of different residents’ mental health status. The first is the gender difference among residents; the independent sample t is used to test whether there are differences in the mental health status of residents of different genders. As a result, the significant probability *p* = 0.19 > 0.05 was obtained. Therefore, gender and positive mental health, and mental distress are not significantly different. However, the positive mental health score of male residents was higher than that of female residents, and the mental distress score of female residents was higher than that of male residents. In general, the mental health status of male residents was higher than that of female residents. The second is the age difference among residents. A one-way analysis of variance (ANOVA) was used to test whether there are differences in mental health status among residents of different ages. The variance difference between various groups of data was compared, and the psychological and emotional changes of residents under the influence of a single control variable were analyzed to evaluate the mental health level of different samples. The results show that the differences in the variance coefficient F value = 13.04, and *p* = 0.03 < 0.05. This shows that there were very obvious differences in positive mental health and mental distress at different ages. A Student—Newman—Keuls test was performed to further study the differences in the age of residents. The results show that the mental distress of residents aged 30–44 and 60–74was quite different. The third is the difference in educational levels. This study used a one-way ANOVA to test whether there were differences in mental health status among different educational levels. The results show that the differences in the variance coefficient between the educational background and mental health status of the residents surveyed is F value = 0.406, and *p*-value = 0.842 > 0.05, indicating that there were no obvious differences in the variance between them. The fourth is the average daily exercise time. In this study, the one-way ANOVA test was used to study whether there was a difference in mental health status during different daily exercise times. The results suggest that F value = 4.70, and *p*-value = 0.02 < 0.05, indicating that there was a significant difference in the variance between the two variables. There are obvious differences in both positive mental health and mental distress of residents in different daily exercise times. It is concluded that the positive mental and emotional situations of those with an average daily exercise time of fewer than 30 min and more than 2 h are significantly different through the Student—Newman—Keuls test.

### 4.5. Multi-Level Multiple Linear Regression Results

**Figure 5:**
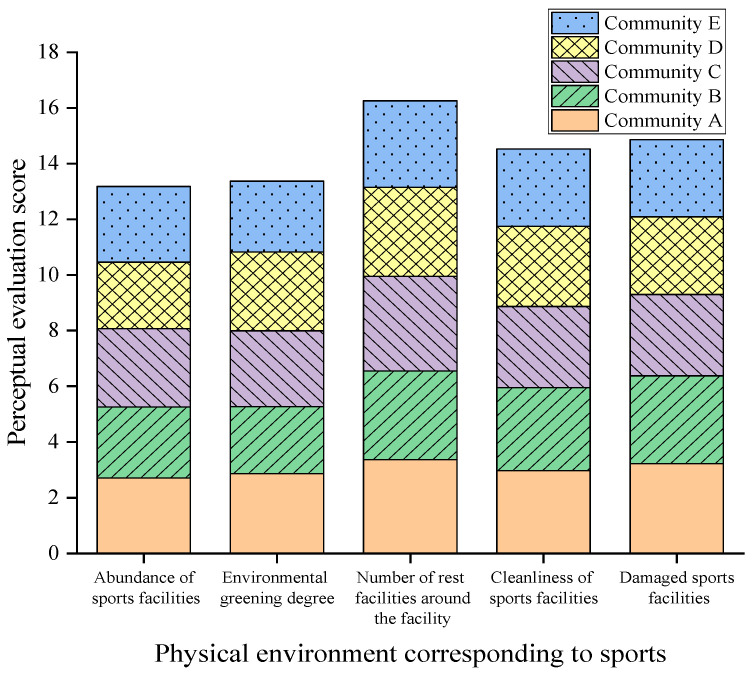
The physical environment perception evaluation score of each community.

A multi-level multiple linear regression model was constructed for the dependent variables of positive mental health and mental distress. A Pearson correlation test and analysis were performed on the activity environment around each community sports facility. A median value of 0.15 and a maximum value of 0.47 were obtained, indicating that the correlation between these independent variables does not lead to serious multi-collinearity problems. In addition, Pearson’s correlation analysis was carried out to analyze the activity environment around sports facilities with positive mental health and mental distress, respectively. The results in Figure 5 show the perceived evaluation scores of the five communities for the material environment that affects residents’ positive mental health and psychological distress. The statistical indicators showed that the availability of sports facilities, the greening of the environment, and the number of rest facilities around the residential area have an important effect on the mental health of residents. The availability of sports facilities and the increase in environmental greening can alleviate negative emotions, and the increase of rest facilities around residential areas can promote the improvement of residents’ mental health levels. It can be seen that the availability of sports equipment and the number of community sports facilities are diverse in different communities, but all communities can maintain a certain average level of cleanliness of sports facilities. The main factor that affects residents’ positive mental health was the cleanliness of sports facilities, while the street environment that affects psychological distress was mainly the degree of damage to sports facilities.

**Figure 6:**
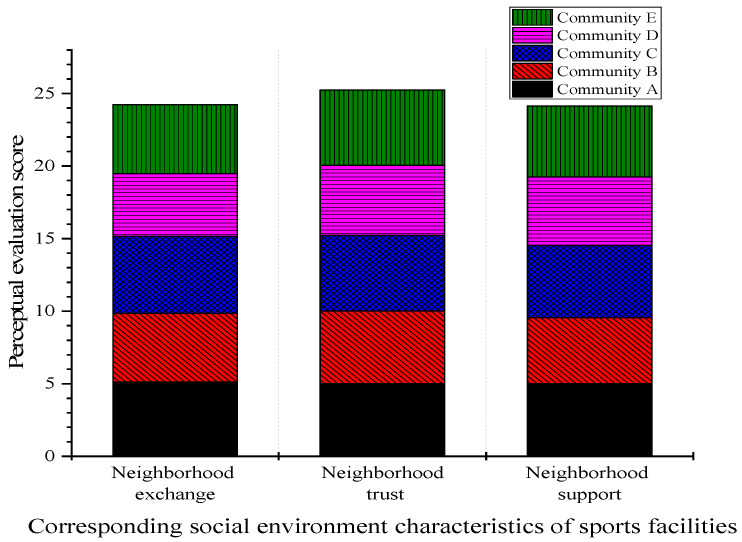
Evaluation scores of social environment perception in each community.

The perceptual evaluation scores of each community for the three social environment characteristics are shown in Figure 6. The figure indicates that neighborhood communication, trust, and support can effectively increase residents’ positive mental health and reduce psychological distress. Neighborhood communication had the greatest effect on improving residents’ mental health. Neighborhood trust had the greatest effect on reducing psychological distress. The results of the regression coefficient demonstrate that the social environment characteristics of sports facilities corresponding to residents’ psychology are mainly neighborhood communication, neighborhood trust, and neighborhood support. Among these three characteristics, the perceived evaluation score of the neighborhood trust factor is relatively high. However, the overall analysis shows that the social environment has less impact on residents than the physical environment.

### 4.6. Mediation Analysis Results

**Figure 7:**
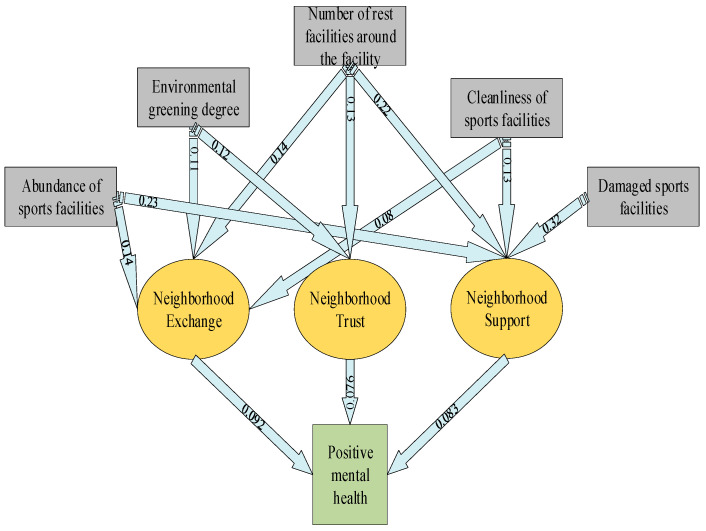
The situation in which social environment characteristics play mediating role in the impact of the physical environment on mental health.

Furthermore, three characteristics of the perceived social environment play a mediating role in the impact of the physical environment on mental health, and their effects are demonstrated in Figure 7. Figure 7 implies that three characteristics of the social environment play a mediating role, but the mediating effect value is small. The number of rest facilities around sports facilities, the availability of sports facilities, and the degree of environmental greening all affect the positive mental health of residents by affecting a certain feature of the perceived social environment. The cleanliness of sports facilities directly impacts the level of positive mental health. It was observed that residents’ mental health includes many influencing factors, among which sports facilities, green environment and the cleanliness of the surrounding environment have a vital effect on residents’ mental health. These factors further constitute a unified mental health evaluation system by adding influences on neighborhood communication, neighborhood trust, and neighborhood support. In addition, the degree of damage to sports facilities directly impacts psychological distress.

### 4.7. Discussions

The reliability coefficient of scale was used in this study to measure the consistency of a measurement scale, and Pearson correlation coefficient was employed to compare the correlation of different items in the questionnaire. The values of the coefficient are always between −1.0 and 1.0, variables close to 0 are known to be uncorrelated, and variables close to 1 or −1 are considered strongly correlated. Through the Pearson correlation coefficient, the correlation degree of different sample questionnaires can be measured statistically, and processed. The Pearson correlation examined the activity environment around sports facilities for positive mental health and psychological distress, respectively. The results demonstrate that 74.9% of the respondents have a mental health risk higher than the threshold, and 26.87% of the respondents have a high level of negative emotions. In addition, neighborhood communication, trust, and support can effectively increase residents’ positive mental health and alleviate psychological distress. Neighborhood communication and neighborhood trust have the greatest effect on improving residents’ mental health and reducing psychological distress. The research results of this study were compared with previous results. For example, Wang et al. [38] studied the mental health care and mental health of the elderly during the COVID-19 epidemic and analyzed the possible clinical characteristics of people with mental health problems. The research suggested that the social consequences of the COVID-19 outbreak may put older adults with pre-existing mental disorders at risk of relapse. Therefore, attention should be paid to the mental health assessment of the elderly, and timely intervention should be carried out for the problem population.

Furthermore, Hirsch et al. [39] evaluated the mental health of residents in specific communities and explored the impact of hydraulic fracturing technology on public mental health by analyzing the process of natural gas exploitation. The results signified that when industry impinges on community life, entire communities can experience collective psychological trauma. Therefore, attention has been paid to the psychological problems of special groups in the community. To sum up, the construction of the current community mental health service system is not satisfactory, and the community mental health service work needs to be further strengthened. Prior studies also suggest that it is necessary to enhance grassroots research [40,41]. Construct the three-level prevention and intervention system of community mental health services, and improve the level of residents’ mental health services.

## 5. Conclusions and Policy Recommendations

This study focuses on the mental health of residents in the community. Data were obtained through a questionnaire survey of nearly 180 residents in five communities to explore the relationship between community public sports services and residents’ mental health. First, mental health was quantified as positive mental health and mental distress through related theories. Second, multi-level regression analysis was used to identify the multiple physical environment characteristics that affect residents’ positive mental health and multiple physical environment characteristics that affect the risk of mental distress-related diseases. Moreover, an improved mediation model was used to verify the mediating role of different social environment characteristics in health effects. Finally, the factors affecting the social-mental process and mental health outcomes were analyzed, and policy recommendations for community public sports services were proposed. This study indicates that the availability of sports facilities, the degree of greening and the increase of the number of rest facilities around the residential environment can ease the mental pain of residents, and improves the mental health level of residents. Furthermore, the cleanliness of sports facilities was found to have a significant positive impact on residents’ mental health. The street environment affects mental health and is attributed to the damage to sports facilities. Neighborhood communication also improves residents’ mental health, and neighbor trust has the greatest effect on reducing mental distress.

Based on the above findings, the study proposes that the overall community should increase the provision of public sports facilities and strengthen the maintenance, care, and cleaning of the facilities and the community environment. A sports organization in the community should be established, and various activities in moderation should be held. Moreover, the publicity of public sports information should be strengthened, and the benefits and precautions of various sports in the community should be given guidance. The community cultural atmosphere should be created, and the residents should be organized to give lectures on fitness. Furthermore, physical monitoring indicators, exercise ability tests, and mental health consultations for residents should be regularly conducted, especially for the elderly. Then, residents can recognize their real physical and mental health status and prevent physical harm caused by exercise. Finally, relevant government departments should do a good job of monitoring and supervision, handle the problems existing in the development of community public sports services in a timely manner, and ensure the normal development of community public sports services.

The research sample is representative, but the number is still small due to the limited research time and article length, which may cause errors in the research results. However, the study has research created paths for future researchers to explore the phenomenon both at home and abroad, which can contribute to improving the physical and mental health of not only Chinese community residents but also people belonging to other geographical settings.

## Figures and Tables

**Table 1:** Current status of supply of community public sports facilities.

Community Public Sports Facilities	General Fitness Facility	Basketball Court	Table Tennis Court	Badminton Court	Indoor Gym	Other Fitness Facilities
Number of communities with facilities	5	4	3	4	2	4
Percentage of communities with facilities (%)	100	80	60	80	40	80

**Table 2:** Current status of supply of community public sports activities.

The Scale of Community Organization Activities	Less than 50 People	50–100 People	100–150 People	150–200 People	More than 200 People
Community A	10	6	3	4	2
Community B	8	5	5	5	1
Community C	11	5	6	3	0
Community D	7	8	6	3	2
Community E	9	6	7	4	3

## Data Availability

The study data will be available on request from authors.
